# Global action for training in malaria elimination

**DOI:** 10.1186/s12936-018-2199-3

**Published:** 2018-01-25

**Authors:** Dyann F. Wirth, Núria Casamitjana, Marcel Tanner, Michael R. Reich

**Affiliations:** 1 Department of Immunology and Infectious Diseases, Harvard T.H. Chan School of Public Health, 665 Huntington Avenue, Building 1, Room 705, Boston, MA 02115 USA; 2 Barcelona Institute for Global Health, Hospital Clínic-Universitat de Barcelona, Carrer Rosselló 132, 08036 Barcelona, Spain; 3 Swiss Tropical & Public Health Institute, Socinstrasse 57, P.O. Box, 4002, Basel, Switzerland; 4 Department of Global Health and Population, Harvard T.H. Chan School of Public Health, Boston, USA

**Keywords:** Malaria, Mosquitoes, Leadership development, Training, Infectious diseases, Human resource capacity

## Abstract

The Rethinking Malaria Leadership Forum, held at Harvard Business School in February 2017 with collaboration of the Barcelona Institute for Global Health and the Swiss Tropical and Public Health Institute, identified this training gap as a high priority for both analysis and action. The gap in human resource training for malaria elimination needs to be addressed in order to assure continued progress. This paper identifies major gaps in skills and human resources, suggests institutions that can assist in filling the training gaps, and proposes global actions to implement expanded training for malaria elimination in endemic countries.

## Background

Since the call for a revitalized global effort to tackle malaria in the past decade, the world has made significant progress toward elimination—but many challenges remain. Malaria elimination has become a high priority goal for many countries, and the strategies to achieve this goal are evolving as both successes and challenges emerged [1, 2]. One core lesson is that the path to elimination is a continuum from control of the disease (including the prevention of death) to the documentation of zero infections and no local transmission [3]. A key need going forward will be a workforce that can rapidly assess failures and success and adapt to the changing malaria landscape. The existing human resources are insufficient in quantity and lack training in how to adapt their approaches as the malaria situation evolves. Previous approaches to training have focused on a set of tools to be implemented rather than a problem to be solved [4]. This shift in emphasis for the malaria workforce requires a new kind of leadership development and training focusing on analytical and problem-solving skills and on informed leadership abilities. Significant investment in training will be required to achieve the global goal of malaria elimination.

## Four training gaps for malaria elimination

Moving toward the global goal of malaria elimination will require major efforts to address four critical gaps in training: front-line field workers, entomologists, research scientists, and malaria-sensitive health system managers, policymakers, and leaders.

Malaria elimination depends on *front*-*line field workers* who will take various actions in controlling the mosquito vector, preventing human contact with infected mosquitoes, assuring the availability and effective use of malaria diagnostic tools, and providing malaria treatment to infected persons. Managing this complex portfolio of malaria interventions in low-resource settings is a major challenge in malaria-endemic countries. Front-line workers need to be trained in how to use the different kinds of technologies in changing disease settings: bed nets treated with long-lasting insecticides, indoor residual spraying, larvicides to use on mosquito breeding sites, diagnostics to determine whether someone is infected with the parasite, and the right medicines to treat infected people. Moving toward malaria elimination requires the use of these technologies, combined in different packages depending on the local transmission conditions and able to respond to changing conditions. Effective training of front-line workers is the first major challenge confronted by all malaria endemic countries; without it, progress will be elusive.

The second major gap in malaria training is *entomologists*—people who understand the biology and behaviour of mosquitos. Basic research is needed to understand the emergence of mosquito resistance to insecticides, but also to understand how the vector may respond to different interventions. Research is needed, for example, on when and where a particular mosquito is most susceptible to spraying, how a particular mosquito might respond to genetically modified mosquitoes, species diversity issues (e.g., responsiveness to interventions, outdoor biting, sensitivity to endectocides, vector capacity). Global warming may change the environments where mosquitoes live and alter the environmental susceptibility to malaria [5, 6]. Field-based and applied research on mosquitoes is also needed to translate basic knowledge into practical information that can be used in adjusting the strategies for malaria elimination in a specific environment. Various training courses have been organized on medical entomology and vector control by the World Health Organization (WHO) [7]. These courses need to be digitalized to make them easily accessible globally and effective in training the expanded work force that is needed to help malaria endemic countries move toward elimination. This training gap is not limited to malaria, but affects the entire field of vector borne-diseases; the recent WHO Global Vector Control Response [2] identifies this gap as a key deficit.

The third gap is in *research training*: fundamental biological research, data science and applied tool development including drugs, vaccines and diagnostics, epidemiological research, and policy research. It will be critical for countries to have a cadre of researchers who can make fundamental discoveries, rapidly integrate new ideas, and use this knowledge to better inform decisions and policy-making, both short- and long-term. There is great opportunity in developing a network of researchers across the globe for contributions to knowledge generation and dissemination. The potential for innovation is greatest when researchers with different perspectives and backgrounds come together to solve a complex problem. The changing malaria landscape presents opportunities for innovation across multiple disciplines—from developing new tools to revisiting the design of clinical trial designs as the malaria endgame nears. A comprehensive research agenda has been developed by the Malaria Eradication Research Agenda (malERA) Refresh Process and this provides a framework for research training [8].

The fourth major training gap for malaria elimination is for *managers and leaders in national health systems*. Health managers responsible for multiple districts, at the regional or state level, need mechanisms to keep track of progress in malaria elimination and algorithms for adjusting control strategies to changing conditions. Smart phones offer an opportunity to create information collection and analysis systems that can provide summary information, analysis, and new data visualization and mapping tools for managers. Policy-makers at the national level will require training in how to monitor and evaluate progress and make adjustments in the package of malaria interventions as conditions change. In addition, managers and leaders need to design and implement effective mechanisms in government agencies to motivate and supervise the front-line workers who will do the work of malaria elimination, and provide them with a reliable supply chain of the needed technologies: bed nets, insecticides, diagnostic kits, and medication. These challenges are features of the health system writ large, but managers and leaders in national health systems will face extraordinary complexities as a result of malaria innovations and transitions in malaria strategy, and products. Solving these challenges will require focused and consistent hard work to make public systems work effectively and responsibly, especially in the difficult organizational environments often found in malaria endemic countries [9].

These four gaps in training for malaria elimination demonstrate the need for a trans-disciplinary approach. Moving forward with malaria elimination requires an understanding not only of human behaviour (for example, how psychology and economics affect the use of nets or the use of health services for diagnosis and treatment) but also mosquito behaviour (for example, which mosquitoes are most susceptible to indoor spraying and how they might be replaced by other mosquitoes that bite outside and during the day) and organizational behaviour (for example, how to manage partnerships between the public and private sectors or across different government ministries). Analysing the costs of different malaria interventions, and determining how to finance those expenditures from domestic and external funds, require both analytical and financial management skills. Training the right people in these skills will draw on a mixture of basic sciences, social sciences, and policy sciences—as often happens in public health.

## Who can deliver the training?

Who can deliver this training for malaria elimination—required for front-line workers, entomologists, researchers, and health system managers and leaders? The training needs to be based in the most recent global knowledge about malaria, delivered with effective pedagogy for adult learners, for both new entrants into the field and existing malaria professionals. The training needs to be mentored and on-the-job training, so that it advances effective implementation rather than interrupts it. Filling these training gaps is not a simple action of “knowledge transfer”. It requires engaging with existing public health professionals in malaria endemic countries and enticing young people to join the struggle against malaria.

One proposal that emerged at the *Rethinking Malaria Leadership Forum* was to create a global network of academic institutions to combine North–South and South–South cooperation strategies. The network could create a shared curriculum of what it will take to achieve malaria eradication on a global scale. The training materials would build on the “Genes to the Globe” approach of Harvard’s Defeating Malaria: From the Genes to the Globe Initiative, which emphasizes a combination of basic science, public policy, and effective implementation. A foundational course could made be available at the global level through an on-line mechanism, and countries could then adapt the basic and more advanced materials to their specific national circumstances. Training needs to be adjusted to the level of each particular participant and their level of decision-making, whether it is front-line, regional, national, or global. Different skills and information are required to make the difficult decisions at each level. The specific training for a country or region would also be dependent on the level of malaria transmission and the kinds of interventions required.

## Actions to expand training for malaria elimination

Training for malaria eradication and elimination will require a global commitment to create a foundational research capacity around the world and to translate new knowledge into actionable policies and programs at the national level. The process of global malaria eradication is likely to extend over the next three to four decades; and the strategies and skills over that time period will need to be adjusted to changing conditions. For example, one of the national success stories of malaria elimination—Sri Lanka—invested in research capacity over a period of 55 years to accomplish malaria elimination [10]. In the process, when malaria resurgence occurred, Sri Lanka benefited from decades of research experiences of malariologists in designing new control strategies that could address changed conditions.

The three institutions represented by the coauthors of this paper are leaders in malaria knowledge generation, translation, and utilization. Over the past 6 years, this team has collaborated in the organization and delivery of an annual leadership development course on the *Science of Eradication: Malaria*. This course provides a strong basis for the foundational knowledge needed to move forward on global mobilization for training in malaria elimination. Additionally the recent launch of a new online course presents some of the core knowledge in advancing the global fight against malaria, in a free online course called (PH425x) *MalariaX: Defeating Malaria from the Genes to the Globe* [11] on the edX online learning platform. This represents a step forward, and many others have resources and capacities to offer, but much more needs to be done.

## Conclusion

Experience in training for malaria elimination, and the gaps in training identified above, informs this proposal of the following three global actions:

1. *Create a group of people* to analyse the scope and scale of the training gaps for front-line workers, entomologists, basic and applied research and malaria-sensitive managers and leaders in health systems, under the auspices of the World Health Organization, with the participation of other organizations such as the Roll Back Malaria Partnership and The Global Fund to Fight AIDS, Tuberculosis and Malaria, as well as bilateral aid agencies. This group could provide concrete estimates of the existing human resources for malaria elimination and of the training needs.
2. *Expand financial support* for training for malaria elimination, based on the expert committee’s analysis, with the participation of government agencies (such as Fogarty International Center at the National Institutes of Health), private foundations (such as the Wellcome Trust and the Bill & Melinda Gates Foundation), and multilateral organizations (such as the Special Programme for Research and Training in Tropical Diseases, better known as TDR).
3. *Create new networks for training* for malaria elimination, involving universities and research institutes in malaria endemic countries along with the implementing government agencies, using the latest in electronic training methods and adult-oriented pedagogy. The training networks would connect new knowledge to field practice in ways that would advance the theory and experience of malaria elimination.

Malaria is a global problem. The world has made remarkable progress in controlling this terrible disease over the past 15 years, pushing down malaria mortality rates globally by 60% from 2000 to 2015, and for children under 5 by 65% [1, p. 6]. The WHO reports a 37% decline in malaria incidence (the rate of new cases) for the same time period. Continuing to make progress toward elimination will require the development of global public goods in training programmes for people at various levels in the health systems of malaria-endemic countries and the implementation of these programmes in the countries with ongoing transmission. Addressing the gaps in global and national training for malaria elimination is achievable, as demonstrated by current collaboration between Boston, Barcelona, and Basel, with the right design, resources, and commitment.

## Abbreviations

malERA: Malaria Eradication Research Agenda (malERA) Refresh Process
TDR: Special Programme for Research and Training in Tropical Diseases
WHO: World Health Organization
